# Kyste du cholédoque rompu: revue de la littérature

**DOI:** 10.11604/pamj.2019.33.276.14372

**Published:** 2019-07-30

**Authors:** Ahmed Azahouani, Najlae Zaari, Fatine El Aissaoui, Mohamed Hida, Mohamed Fitri, Larbi Benradi, Houssain Benhaddou

**Affiliations:** 1 Service de Chirurgie Pédiatrique, CHU Mohamed VI, Oujda, Maroc

**Keywords:** Rupture, kyste du cholédoque, voies biliaires, Rupture, cyst of the common bile duct, biliary tract

## Abstract

Les malformations kystiques des voies biliaires sont des affections congénitales rares évaluées à environ 1/2 000 000 naissances. C'est une affection qui peut se révéler gravement par une complication notamment l'angiocholite, la pancréatite chronique, cirrhose biliaire progressive, l'hypertension portale ou les lithiases biliaires. Sa perforation spontanée est l'une des rares complications, décrite pour la première fois en 1934 par Weber. Nous rapportant le cas d'un garçon de 18 mois admis pour syndrome sub-occlusif avec une péritonite biliaire. Une échographie a été réalisée montrant un épanchement abdominal avec formation kystique communicante des voies biliaires associée à un épanchement sous capsulaire du foie confirmé par scanner. L'intervention a consisté en une toilette péritonéale avec mise en place d'un drain de redon au niveau de la perforation et un drain sous hépatique sans excision du kyste. Le patient a été réadmis 6 mois après cet incident pour sa cure définitive.

## Abstract (en)

Cystic malformations of the bile ducts are rare congenital disorders, with an incidence of 1/2000000 live births. Complication including the angiocholitis, chronic pancreatitis, progressive biliary cirrhosis, portal hypertension or gallbladder lithiases can reveal severe disorder. Spontaneous perforation is one of the rare complications described for the first time in 1934 by Weber. We report the case of an 18-month old baby admitted with subocclusive syndrome with biliary peritonitis. Ultrasound was performed showing abdominal effusion with cystic formation communicating with the bile ducts associated with subcapsular effusion of the liver confirmed by a scanner. Treatment was based on peritoneal toilet with redon drain at the level of the perforation and subhepatic drain without cyst excision. The patient was re-admitted 6 months after this incident to be definitively treated.

## Introduction

Les malformations kystiques des voies biliaires sont des affections congénitales rares [1]. L'étiologie la plus communément admise est l'anomalie de la jonction bilio-pancréatique qui est aussi incriminé dans la dégénérescence des voies biliaires. L'age de survenue de cette malformation est moins de 10 ans avec une prédominance feminine [2]. La triade douleur, ictère, masse évoque classiquement une dilatation kystique des voies biliaires or il n'est retrouvé que dans 13 à 25% des cas [3]. Exceptionnellement cette affection peut se révélé gravement par une complication. La résection de la dilatation kystique est le traitement de référence. L'anastomose kystodigestive est actuellement abandonnée car la malformation kystique des voies biliaires est considérée comme un état précancéreux [2].

## Patient et observation

**Figure 1:**
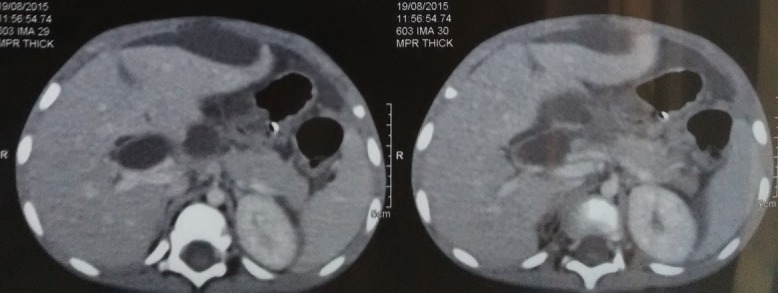
Aspect de la rupture du kyste du cholédoque sur une TDM

**Figure 2:**
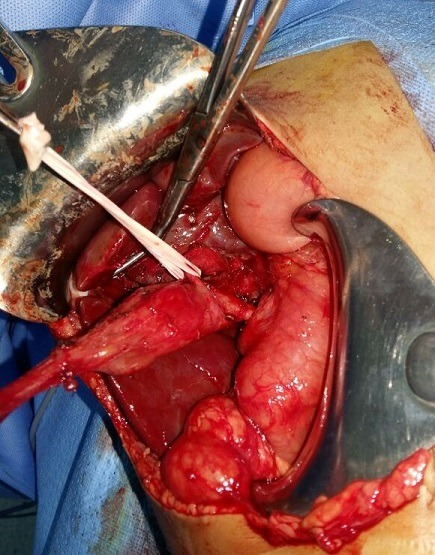
Aspect de la dilatation kystique en peropératoire

**Figure 3:**
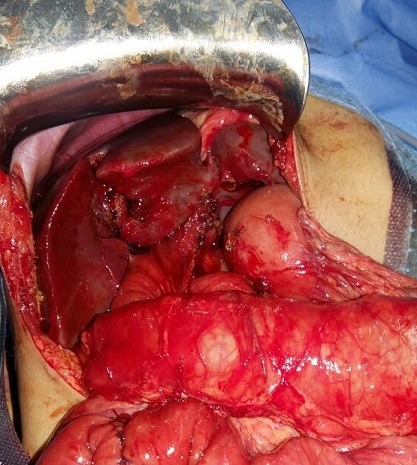
Anastomose cholédocho jéjunale en Y

Un garçon de 18 mois sans antécédents pathologiques particuliers qui a été admis aux urgences pédiatriques pour des vomissements alimentaires depuis 7 jours, devenant bilieux récemment, des cris, et fièvre. L'examen clinique à l'admission montrait une température à 38.5°C, un abdomen ballonné, avec une sensibilité abdominale diffuse. L'état général était conservé en dehors d'une asthénie, l'état hémodynamique était stable, sanssignes de choc. Un abdomen sans préparation a été réalisé revenu sans anomalies. Une échographie faite avait montré un épanchement abdominal avec formation kystique communicante des voies biliaires associée à un épanchement sous hépatique. Une ponction hépatique échoguidée a été réalisée affirmant l'origine bilieux du liquide sous hépatique. Le complément scanographique confirmait les données de l'échographie (Figure 1). Les marqueurs biologiques étaient en faveur d'un syndrome inflammatoire modéré et les enzymes hépatiques étaient normales. Une laparotomie sous costale droite a été réalisée pour suspicion de rupture du kyste du cholédoque. L'exploration chirurgicale a confirmé la présence d'une péritonite biliaire non purulente, liée à la perforation d'un kyste du cholédoque. L'intervention chirurgicale initiale a consisté en une toilette péritonéale avec mise en place d'un drain de redon au niveau de la perforation et un drain en sous hépatique sans excision du kyste. Le foie était d'aspect normal sans signes de cirrhose. Les suites postopératoires ont été simples et l'enfant a pu quitter le service au dixième jour postopératoire. Le patient a été réadmis 6 mois après cette incident pour cure définitive de sa malformation avec réalisation d'une résection complète large de la dilatation de la voie biliaire principale, avec anastomose hépatico jéjunale sur une anse montée en Y selon Roux (Figure 2, Figure 3).

## Discussion

La première description de la dilatation canalaire de l'arbre biliaire date de 1723 par Vater [4]. C'est une malformation rare, qui se voit volontiers chez l'enfant (75% des cas sont découverts avant l'âge de dix ans), avec une nette prédominance féminine (sex-ratio entre 0,23 à 0,43) [2]. Son incidence est de l'ordre de 1/2 000 000 naissances [1]. Elle se voit surtout dans les pays asiatiques [5]. Bien que touchant préférentiellement l'enfant, le kyste du cholédoque (DKC) ne peut se manifester qu'à l'âge adulte, sous la forme d'une complication, principalement infectieuse [6]. L'hypothèse étiologique la plus communément proposé étant que le kyste du cholédoque est le résultat d'une anomalie de la jonction pancréatico biliaire [7]. Récemment, un fonctionnement anormal du sphincter d'Oddi a été rapporté comme prédisposant au reflux de suc pancréatique dans la voie biliaire, et des spasmes du sphincter d'Oddi ont été notés en association aux kystes du cholédoque [8]. La classification de Todani est la plus utilisée et comprend cinq types [7], le type le plus commun est le type I, correspondant à une dilatation de la voie biliaire extra-hépatique, et divisé en 3 sous-types: Ia = dilatation kystique, Ib = dilatation segmentaire et Ic = dilatation fusiforme [7].

Le diagnostic peut être évoqué en anténatal devant la constatation d'une formation liquidienne au niveau de la région du hile hépatique [5, 9]. Chez l'enfant sa découverte est fortuite, et quand elle est symptomatique, elle se manifeste par la triade classique: douleur abdominale, ictère, masse de l'hypochondre droit [3]. Cette symptomatologie n'est observée que dans 13 à 25% des cas [3]. La DKC est rarement découverte au stade de complications [10], et qui sont: la lithiase qui peut bloquer le bas cholédoque, l'infection (angiocholite, abcès hépatique ou septicémie) [5], la cirrhose biliaire primitive, l'hypertension portale, lacholangite et la dégénérescence maligne à type de cholangiocarcinome avec une fréquence variable en fonction de l'âge du patient, de l'ordre de 0,7% moins de dix ans et 6,8% entre 11 et 20 ans et beaucoup plus élevée chez l'adulte [2, 11]. La rupture spontanée est une de ces rares complications. Son incidence est de 1,8% à 2% [3]. Dans la majorité des cas elle survient chez les enfants âgés de moins de 4 ans [3] et elle a été décrite pour la première fois en 1934 par Weber [3]. Le tableau clinique dans ce cas est habituellement une distension abdominale progressive, vomissements, et état de choc avec ou sans ictère [3]. La rupture traumatique est encore plus rare, quelques cas seulement ayant été décrits [5, 12].

En échographie, la DKC se présente comme une masse kystique appendue ou remplaçant le cholédoque et se prolongeant en haut avec le canal cystique et le canal hépatique, et en bas avec le cholédoque terminal ou le canal de Wirsung [3]. Cet examen peut suffire au diagnostic et la TDM n'apporte pas d'informations supplémentaires [3, 10]. La cholangio-TDM permet de visualiser l'accumulation du produit decontraste dans le kyste. Une cartographie de l'arbre biliaire et une délimitation précise de la lésion sont ainsi obtenues [10]. La cholangio IRM ou bili-IRM est une technique récente et non invasive très performante dans le diagnostic des anomalies de la jonction bilio-pancréatique [3]. Dès que le diagnostic d'une dilatation kystique du cholédoque est posé, il convient de faire la cure chirurgicale à l'âge de 6 mois [13]. L'opération chirurgicale la plus communément admise consiste à réséquer toute la portion dilatée de la voie biliaire et réaliser une anastomose cholédocho jéjunale ou hépatico jéjunale sur une anse en Y à la Roux [3, 5, 14]. La résection est large vu le risque de dégénérescence secondaire de la paroi biliaire dysplasique [3]. Pour les kystes intrahépatiques et les dilatations intrahépatiques (type IV de Todani), d'autres interventions peuvent être nécessaires telle qu'une segmentectomie, une hépatectomie partielle ou bien une kysto entérostomie intrahépatiques [2, 15]. Il est recommandé de faire une biopsie hépatique afin de déceler des signes précoces de cirrhose hépatique [5].

## Conclusion

La rupture d'un kyste du cholédoque est une rare cause d'abdomen aigu chez l'enfant et qui peut évoluer sur à bas bruits pendant quelques jours. Le diagnostic est évoqué par l'échographie et le scanner et confirmé en peropératoire. Le traitement consiste en la toilette péritonéale et la résection du kyste avec une anastomose cholédocho jéjunale en Y.

## Conflits d’intérêts

les auteurs ne déclarent aucun conflit d'intérêts.
